# Obscure Bleeding from a Metastatic Small Bowel Tumor Diagnosed Using Motorized Spiral Enteroscopy: A Case Study and a Literature Review

**DOI:** 10.3390/diagnostics14090904

**Published:** 2024-04-26

**Authors:** Christian Banciu, Andreea Munteanu, Adrian Aprotosoaie, Ramona Fabian, Amadeus Dobrescu, Adrian Vaduva, Antonio Fabian, Irina Soica, Viviana Ivan, Laurentiu Sima

**Affiliations:** 1 Department of Internal Medicine IV, Faculty of Medicine, “Victor Babes” University of Medicine and Pharmacy, 2 Eftimie Murgu, 300041 Timisoara, Romania; banciu.christian@umft.ro (C.B.); adrian.aprotosoaie@gmail.com (A.A.); rgerlinder@yahoo.com (R.F.); 2 Department of Surgery II, Faculty of Medicine, “Victor Babes” University of Medicine and Pharmacy, 2 Eftimie Murgu, 300041 Timisoara, Romania; amadeusdobrescu@yahoo.com; 3 Department of Microscopic Morphology-Morphopatology, “Victor Babes” University of Medicine and Pharmacy, 300041 Timisoara, Romania; vaduva.adrian@umft.ro; 4 ANAPATMOL Research Center, “Victor Babes” University of Medicine and Pharmacy, 300041 Timisoara, Romania; 5 Department of Pathology, “Pius Brinzeu” County Clinical Emergency Hospital, 300723 Timisoara, Romania; 6 Clinical Hospital of Infectious Diseases and Pneumophysiology Dr. Victor Babeș Timișoara, 300310 Timisoara, Romania; antoniofabian9319@yahoo.com; 7 Medical School, University College London, 74 Huntley St., London WC1E 6DE, UK; irina.soica.20@ucl.ac.uk; 8 Department of Cardiology I, Faculty of Medicine, “Victor Babes” University of Medicine and Pharmacy, 2 Eftimie Murgu, 300041 Timisoara, Romania; ivan.viviana@umft.ro; 9 Department of Surgical Semiology I and Thoracic Surgery, Faculty of Medicine, “Victor Babes” University of Medicine and Pharmacy, 2 Eftimie Murgu, 300041 Timisoara, Romania; lica_sima@yahoo.com

**Keywords:** motorized spiral enteroscopy (MSE), small bowel tumors, primary lung adenocarcinoma, obscure bleeding

## Abstract

Small bowel tumors are relatively rare, representing only around 5% of all gastrointestinal neoplasms, with a progressively increasing incidence. Currently, there are no established guidelines for diagnostic approaches, screening procedures, or management strategies for small bowel tumors. We present here the case of a patient with a rare type of metastatic tumor of the small bowel originating from primary lung adenocarcinoma who presented with abdominal pain, severe iron-deficiency anemia, and melena. The initial investigations, gastroscopy and colonoscopy, failed to identify the bleeding source. The obscure bleeding source and diagnosis were achieved through power motorized spiral enteroscopy (MSE), which allowed the visualization and biopsy of the tumor. Histopathological examination established the presence of a poorly differentiated non-mucinous adenocarcinoma originating from the lung. This case is reported to provide evidence of the efficiency of MSE in the diagnosis of small bowel tumors, with the method providing higher insertion depth in a reduced amount of time.

## 1. Introduction

Despite the small bowel accounting for more than 90% of the gastrointestinal surface, it rarely develops primary neoplasms, although the last decade has witnessed an increase in their numbers. [1]. Considering its silent course with non-specific symptoms, its diagnosis and management have proved rather challenging. 

The discovery of a small bowel neoplasm is usually associated with a metastatic process in patients diagnosed with a primary malignancy. Studies revealed that 50% of small bowel malignant tumors are of metastatic nature and originate from the colon, stomach, pancreas, melanoma, breast, or lung [2].

The most common clinical symptoms indicating a small bowel tumor are unexplained iron-deficiency anemia on the background of a negative gastroscopy and colonoscopy, abdominal pain, anorexia, and weight loss [3]. A correct diagnosis requires both strong clinical suspicion and the use of various investigation tools, including endoscopy, which overcomes the challenges that arise from small bowel length and tortuosity. In addition to eso-gastro-duodenoscopy and colonoscopy, power motorized spiral enteroscopy (MSE) allows the direct visualization of the entire small bowel. Although there are two other types of deep-enteroscopy devices, single- and double-balloon types, both safe and effective, they require a significantly longer investigational time. Using the power MSE, a trained clinical investigator manages to visualize the entire small bowel within 45 min–1 h.

MSE was preceded by manual spiral enteroscopy, a procedure introduced in 2008 that consisted of the manual rotation of a spiral over a tube that allowed the folding of the bowel over the enteroscope [4]. Even though it reduced the time of exploration compared to the balloon types, it did not improve the insertion depth. The power MSE procedure employs a similar fundamental mechanism but uses a motorized handle that can be switched by the operator, thus allowing a further reduction of the exploration time while providing a higher insertion depth.

In this paper, we report the case of a patient who presented with abdominal pain, anemia, and melena that prompted a suspicion of obscure small bowel bleeding. A power MSE was subsequently conducted, and the procedure revealed the presence of a bleeding small bowel tumor that was identified as a lung cancer metastasis. The reported incidence of symptomatic gastrointestinal metastasis in lung cancer is very low (0.2–0.5%), being presumably underdiagnosed due to its classification as a generalized metastatic disease or as a side effect of conventional chemotherapy [5]. However, modern therapies have prolonged the average life expectancy, thus prompting a rise in the incidence of intestinal metastasis. Regardless of its frequency, the accurate diagnosis of gastrointestinal metastasis may prevent unnecessary medical procedures and their complications.

## 2. Case Presentation

A 66-year-old male with a past medical history of chronic gastritis, type 2 diabetes mellitus, hypertension, intestinal polyposis, and severe iron deficiency anemia was referred from another hospital to our unit, presenting right hypochondrium abdominal pain, fatigue, nausea, unintentional significant weight loss (15 kg over 3 months), and melena. The hospital discharge papers revealed normal gastroscopy and colonoscopy results and the presence of an obscure hemorrhage. Therefore, taking into account the history of intestinal polyps, the presence of obscure bleeding, and other symptoms, as well as the results obtained from the previous hospital, the patient was referred for MSE. Upon admission to our unit, the physical examination revealed malaise, pallor, dehydration, and a constitutionally overweight status (BMI = 25.2 kg/m^2^). 

Objective examination of the digestive system demonstrated a soft, mobile abdomen with tenderness, spontaneously and upon palpation, in the right hypochondrium region. The presence of melena was affirmed, with no signs of peritoneal irritation. Abdominal ultrasound revealed a normal liver; no space-replacing formations (metastases) or lymphatic nodules in the hepatic hilum or para-aortic nodes were observed. The blood test results upon hospital admission are detailed in Table 1. Due to the low values of serum iron (27 μg/dL) and hemoglobin (9.6 g/dL), the patient received a blood transfusion to correct the severe iron-deficient anemia (Table 1). 

### 2.1. Motorized Power Spiral Enteroscopy

**Figure 1:**
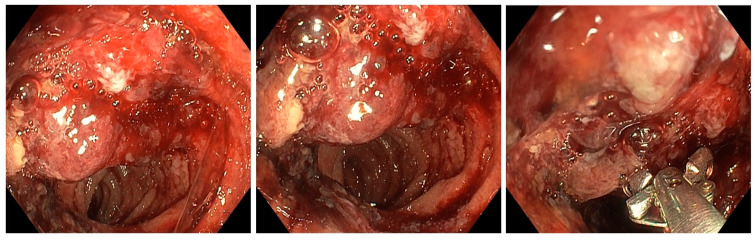
Enteroscopic view showing the bleeding ileal tumor lesion.

The presence of significant weight loss and gastrointestinal symptoms (abdominal pain and melena—indicative of gastrointestinal bleeding), along with a history of chronic gastritis, severe iron deficiency anemia, and other comorbidities, including diabetes and hypertension, prompted the decision to perform an endoscopy. Failure to identify the source of bleeding through gastro- and colonoscopy prompted further examination into the small intestine, which led to the decision to perform an enteroscopy. The source of the bleeding was detected by MSE using a retrograde approach (Figure 1). 

During MSE, the examination revealed no stigmata of large bowel disease. However, in the ileum, a bleeding non-circumferential protrusive-ulcerative lesion, measuring approximately 3–4 cm, was observed at a distance of around 130 cm from the anal verge. Post-lesion, the small intestine mucosa appeared normal. Additionally, at around 140 cm from the anal orifice, a small, flat polyp measuring approximately 0.8–1 cm was observed in the small intestine. Biopsies were taken from both the ileal tumor lesion and the flat intestinal polyp, and India ink was injected to help the surgeon visualize the location of the tumor formation. The maximum insertion depth was 150 cm. 

The ileal tumor lesion biopsies revealed exulcerated intestinal mucosa tissue, indicating the presence of a malignant tumor growth composed of clusters and plaques of medium-sized cells. These cells exhibited ample, clear, or faintly eosinophilic cytoplasm, and vesicular nuclei with pronounced contour irregularities, some of them displaying eosinophilic nucleoli. Additionally, numerous small-sized vessels with active hyperemia were identified. Immunohistochemical analysis was recommended to determine the cellular lineage involved in the proliferation. The biopsy from the polyp revealed tissue sections consisting of intestinal mucosa with edematous corium, along with chronic inflammatory infiltrates displaying diffuse distribution.

### 2.2. Thoracic Computed Tomography 

**Figure 2:**
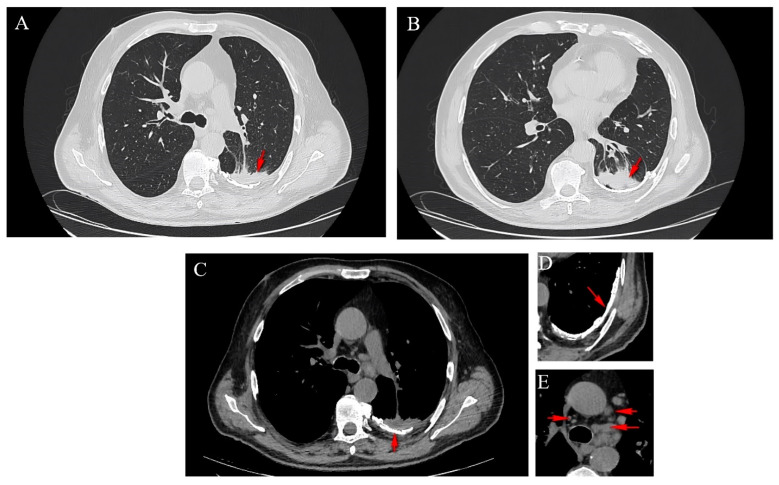
Axial CT sections showing a spiculated lesion in the LSL (**A**), a lesion in the LLL (**B**), the presence of a left-sided calcified pachypleuritis (**C**,**D**) and mediastinal adenopathies (**E**).

A computed tomography (CT) scan of the thorax revealed the presence of a left-sided calcified pachypleuritis, predominantly at the level of the left lower lobe (LLL), chronic pulmonary consolidations without air bronchograms that exhibited connections to the bronchi, with thickened walls in the apical segment of the LLL (approximately 5.5 cm), a consolidation area without air bronchogram in the posterior segment of the left superior lobe (LSL, approximately 5.6 cm), and left mediastinal-hilar adenopathies measuring up to 1.9 cm (Figure 2). No pleural collections or pericardial effusions were present. The large mediastinal vessels presented normal caliber. Other CT findings included calcified parietal atheromatosis of the aorto-coronary artery and degenerative disc-vertebral changes in the thoracic region.

### 2.3. Surgical Procedure and Pathological Findings

**Figure 3:**
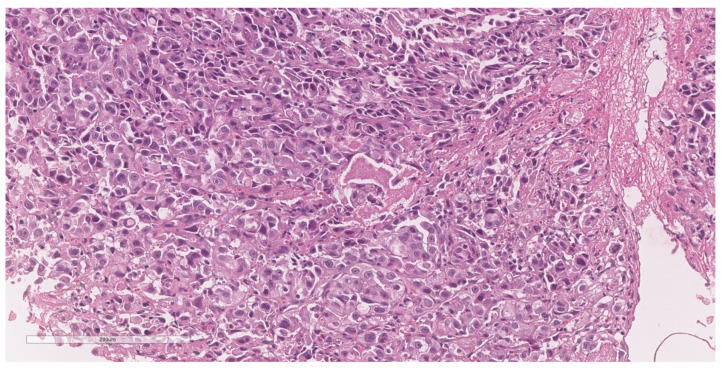
Sample H&E-stained section showing a poorly differentiated malignant tumor, composed of round to cuboidal cells, arranged in solid and trabecular patterns. The nuclei are enlarged, pleomorphic, and vesiculous, with prominent nuclei. Atypical mitoses and necrosis are present (20× magnification).

**Figure 4:**
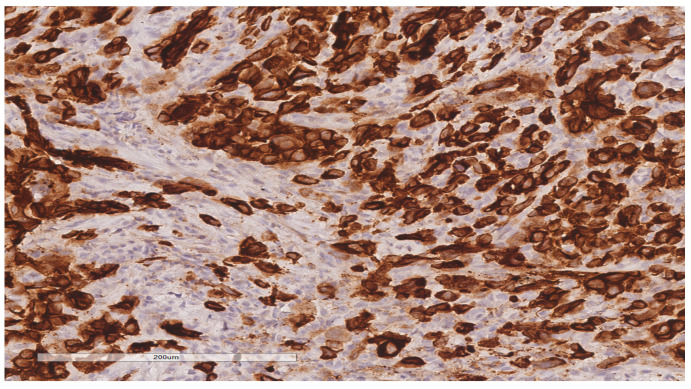
Immunohistochemical reaction for CK7 shows intense, diffuse staining in tumor cells (20× magnification).

**Figure 5:**
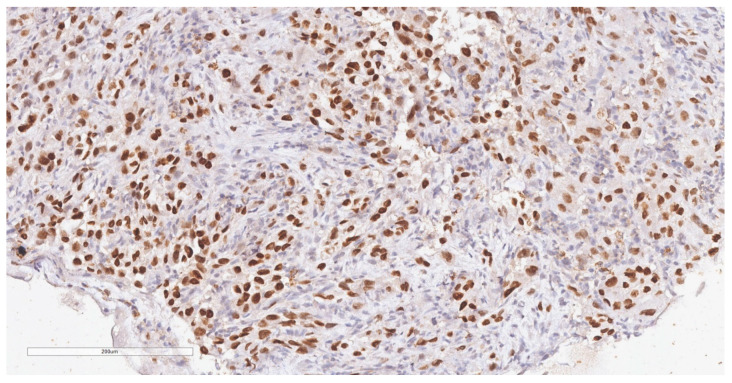
Immunohistochemical reaction for TTF1 shows intense, diffuse, nuclear staining in tumor cells (20× magnification).

The patient was referred to the surgical department and underwent tumor resection. The intraoperative diagnosis was a cT2N2M0 stage IIIB ileal tumor with peritoneal adhesions. A firm, non-serosal-expressing intestinal tumor formation measuring approximately 5/4 cm was identified. A segmental enterectomy was performed over a length of approximately 60 cm. The lymph node block found at the level of the mesentery corresponding to the ileal formation was also removed. The histopathological aspects and immunohistochemical profile of the biopsied fragments, according to hematoxylin and eosin (H&E) staining (Figure 3) and immune reaction with CK7 and TTF1 antibodies (Figure 4 and Figure 5), suggested the presence of multiple metastases (intestinal, mesenteric, lymph nodal) of a poorly differentiated non-mucinous, predominantly solid adenocarcinoma originating from the lung.

## 3. Discussion

Small bowel tumors are rare, representing only around 5% of all gastrointestinal neoplasms, with a progressively increasing incidence of 0.3–2 cases/100,000 individuals; more specifically, metastatic tumors occur more frequently than primary tumors [6]. Due to its nonspecific clinical features, diagnosis is usually delayed by 6–10 months from symptom onset [7], which is reflected in a poor patient prognosis. Additionally, the diagnosis is hampered by the anatomical conditions, which pose a challenge for endoscopic and imaging procedures [1]. Moreover, on suspicion of a small bowel lesion, differentiating between benign and malignant disease is highly dependent on the stage and degree of the condition and therefore cannot be established based on clinical presentation alone. Due to the reduced frequency of small bowel tumors, there are currently no established guidelines for diagnostic approaches, screening procedures, or management strategies.

The patient described in the present case report has a history of intestinal polyposis and presented to our clinical setting with severe anemia diagnosed as iron-deficiency anemia. The presence of melena indicated that the anemia occurred as a consequence of blood loss. The patient presented other nonspecific symptoms such as nausea, burning-type pain in the right hypochondrium, fatigue, and weight loss. Altogether, these signs and symptoms prompted the suspicion of an intestinal lesion of unknown nature that needed to be investigated endoscopically. As esophagogastroduodenoscopy and colonoscopy revealed no specific findings, the investigation of the small bowel was deemed imperative [8]. The power MSE in the retrograde approach was chosen as the endoscopic procedure of choice. 

Gastrointestinal (GI) bleeding can be classified as overt or occult. Overt GI bleeding is visible, presenting hematemesis (blood or coffee-ground-like material vomit), hematochezia (bloody stool passage), or melena (a dark and tarry stool) [9,10]. Conversely, occult or chronic GI bleeding stems from microscopic hemorrhage and often presents as Hemoccult-positive stools, accompanied or not by concurrent iron deficiency anemia [9,10]. Obscure bleeding is defined as overt GI bleeding that originates from an unidentified source during an initial assessment through standard endoscopic or radiographic methods [9,10]. In our case, the patient presented obscure bleeding, characterized by visible hemorrhage manifested as melena, and without identifiable origin using the standard gastroscopy and colonoscopy approaches.

Small bowel endoscopy can be achieved by using a small bowel capsule endoscopy (SBCE) that allows visualization of the entire small intestine but cannot perform biopsies, hemostasis, or endoscopic treatments [11]; moreover, some clinical scenarios (strictures, compressing tumors, polyps) may cause lodging of the SBCE in the small bowel, requiring extraction either through endoscopy (using enteroscopy) or through surgical means. MSE in either the antegrade or retrograde approach was introduced in 2018 as a safe and effective procedure for complete enteroscopy conducted in a short time (~30 min) [12,13]. In contrast to manual spiral enteroscopy, the power motorized version allows deeper insertion in a reduced amount of time and easier maneuvering of the endoscope [12]. The time of exploration is a key element in reducing hospital costs and increasing the number of procedures performed [14]. Although conventional device-assisted enteroscopy procedures such as balloon (single- or double-) enteroscopy stand as effective non-surgical diagnostic and therapeutic tools for small bowel lesions, they require a considerably longer exploration time compared to spiral enteroscopy and involve substantial radiation exposure due to continuous fluoroscopy [4,15]. Moreover, therapeutic balloon enteroscopy exhibits a complication rate ranging from 3.4% to 4.3%, including adverse events such as pancreatitis, perforation, hemorrhage, and balloon dislocation [16]. By comparison, MSE demonstrated a lower incidence of severe complications, with an overall rate of 0.4%, and a perforation rate of 0.34% [17]. A 2023 randomized controlled trial compared MSE to single-balloon enteroscopy, analyzing their total enteroscopy rates (TER) and diagnostic efficacies for small bowel disorders; the results suggest that higher TER values can be achieved by MSE in a shorter period of time with similar, minimal adverse events [18].

Our hospital was the first and only clinical setting in Romania at the time of the case that had the means to perform MSE; it is mainly indicated in patients that require deep enteroscopy for diagnosis or therapeutic purposes, allowing interventions such as argon plasma coagulation, hemoclipping, polypectomy, or stricture dilatation. Conversely, the procedure is not recommended in patients where general anesthesia is contraindicated, patients with suspected perforation or uncontrolled coagulopathy, or pediatric patients; in particular, retrograde enteroscopy cannot be conducted in patients with severe colon inflammation or stricture [15]. In a prospective single-center study that assessed 62 patients by either ante-/retrograde/bidirectional MSE, technical success was achieved in 94% of cases and satisfactory insertion depth in 89% of cases; total enteroscopy was accomplished in 84% of patients with a mean insertion time of 44 min in the retrograde approach [19]. Similar results were reported by Wang et al. [20]; mild adverse events were generally self-limiting or even asymptomatic, while severe adverse events were a rare occurrence (0.7%) and consisted of mild pancreatitis, intussusception of the sigmoid colon during endoscope withdrawal or perforation of the bowel. Although mastering this technique is a steep learning curve, in experienced hands, it proves to be highly beneficial. The range of studies conducted, as referenced in the current case presentation, demonstrate its safety and efficacy conclusively. Endoscopic visualization may be more effective than imaging procedures. In one case report of a patient diagnosed with multiple location metastases and strongly positive results for occult bleeding, the abdominal CT examination did not reveal any obvious signs of tumor or perforation; however, it indicated the presence of free air and ascites in the peritoneal cavity [21]. 

In our case, the procedure revealed a protrusive-ulcerative tumor formation in the small bowel in the jejunum-ileum segment; the histopathological examination established the presence of a poorly differentiated, predominantly solid, non-mucinous adenocarcinoma of the pulmonary starting point, with a single metastasis (intestinal) and regional lymph nodes (mesenteric). Out of the five main types of small bowel tumors, adenocarcinoma is the most frequently encountered, occurring mainly in the duodenum or jejunum [22]. The analysis of 366 cases of lung cancer that metastasized in the gastrointestinal tract revealed the small bowel as the most frequent metastasis location, with adenocarcinoma-type cells among the most frequently identified [23]. However, the study also reported that lung adenocarcinoma shows the lowest risk of gastrointestinal metastasis compared to other types of lung cancer. 

In our patient, the small bowel single metastasis was discovered and biopsied through MSE before any suspicion of a broncho-pulmonary adenocarcinoma. Although gastrointestinal metastasis of lung cancer is not exceptional, it usually progresses without clinical manifestation, potentially leading to bowel perforation. In our case, the patient presented severe anemia consequent to blood loss, melena, and other nonspecific symptoms such as nausea, pain in the right hypochondrium, fatigue, and weight loss. Small bowel metastasis typically indicates advanced-stage lung disease, as it is most frequently discovered incidentally during autopsies [24]. The discovery of bowel metastasis as the initial diagnostic of lung cancer is rare and usually occurs due to abdominal perforation; the report of such cases contradicts older theories that small bowel perforation in lung cancer patients occurs due to the necrotic effect of chemotherapy on cancer cells [25,26]. 

The surgical intervention in small bowel metastasis is a palliative procedure, usually associated with a poor prognosis, often due to septic shock despite an urgent procedure [26]; in several case reports, death occurred within weeks or months post-operatively [27]. However, one case report presented the management of a small bowel metastatic lung cancer patient who underwent surgical removal of the bowel tumor, followed by oral targeted therapy; the patient’s last follow-up was conducted 6 months after the intervention, indicating that early detection and diagnosis combined with effective treatment may prolong patient survival beyond 6 months [28]. Regarding our case, the MSE intervention proved efficacious, devoid of any adverse effects. This technique effectively stopped the bleeding and permitted the acquisition of a biopsy, hence enabling the later surgical intervention. Following the surgical procedure, which involved segmental enterectomy spanning approximately 60 cm, excision of the mesenteric lymph node block corresponding to the ileal formation, and subsequent histopathological analysis, the patient underwent chemotherapy. As of the 18-month postoperative follow-up, no evidence of recurrent disease was reported; the patient is currently undergoing chemotherapy for the lung carcinoma. The early utilization of MSE may prevent the need for unnecessary medical procedures and their associated complications, thereby expediting diagnosis and intervention in cases of obscure gastrointestinal bleeding.

Similarly to our case, Ying et al. presented the case of a patient previously diagnosed with primary solid subtype lung adenocarcinoma who developed anemia and melena, being subsequently diagnosed with small intestine and mesenteric lymph node metastasis; following abdominal surgery, the patient survived for approximately 6 months [29]. Therefore, in cases with anemia and melena, enteric metastasis should be considered as part of the differential diagnosis, particularly in patients with a previous diagnosis of lung cancer [30].

Even more similar to our case, Li et al. reported the case of one patient whose only symptoms were abdominal distention and discomfort and who was diagnosed with gastric and small bowel tumors through CT scans; five examinations of stained smears of sputum were needed to diagnose the presence of non-resectable squamous-cell lung cancer [31]. Intestinal obstruction was the primary symptom in a case of lung cancer reported by Janez in 2017 [32]; diffuse metastatic lesions were present along the entire length of the small bowel and were identified as poorly differentiated adenocarcinomas originating from primary lung cancer.

Despite potential limitations in accessibility in certain healthcare settings and the requirement for considerable specialized training, competency, and adherence to safety regulations, the use of MSE offers notable advantages. MSE enables a real-time prompt examination and intervention in the small bowel with a low risk of complications, hence expediting the diagnosis and management of bleeding lesions. This technique is particularly valuable in situations where conventional gastro- and colonoscopy are unable to detect the cause of bleeding, as demonstrated in our case. When patients experience abdominal pain, anemia, and melena, it is important to consider enteric metastases as a potential diagnosis, particularly in patients with lung cancer. The consideration of MSE in the management of bleeding episodes is recommended as it has the potential to enhance patient outcomes and improve overall health-related quality of life.

## 4. Conclusions

In patients with lung cancer, the possibility of enteric metastasis is warranted. An early, accurate diagnosis of gastrointestinal metastases, as provided by MES, may prevent unnecessary medical procedures and can enable a timely intervention that will optimize patients’ treatment and quality of life. Our experience demonstrated the effectiveness of MSE in detecting a small bowel lesion that was missed by conventional procedures. Although MSE requires specialized skills and carries procedural risks, it presents notable benefits such as immediate evaluation and intervention, along with a minimal rate of complications. In our case, the MSE intervention proved to be highly efficient in bleeding arrest and biopsy retrieval without producing any adverse effects. This technique proved to play a crucial role in diagnostic and decision management, ultimately improving patient outcomes and overall quality of life. Consequently, MSE should be incorporated into the diagnostic algorithm of patients with abdominal pain, anemia, and melena, where a metastatic disease is suspected, to optimize patient care and treatment outcomes.

## Figures and Tables

**Table 1:** Blood test results.

	Values	Reference Values
Erythrocytes (RBC)	3.69 × 10^6^/μL	4.5∙10^6^–5.9 × 10^6^/μL
Hemoglobin (HGB)	9.6 g/dL	13.5–17.5 g/dL
Hematocrit (HCT)	30.60%	37–53%
Mean corpuscular hemoglobin concentration (MCHC)	31.4 g/dL	32–36.5 g/dL
Leukocytes	7.6 × 10^3^/μL	4.5 × 10^3^–10 × 10^3^/μL
Lymphocytes	22%	24–44%
Thrombocytes (PLT)	494 × 10^3^/μL	150 × 10^3^–450 × 10^3^/μL
Erythrocyte Sedimentation Rate (ESR)	57 mm/h	1–15 mm/h
INR	1.11 INR ISI	0.9–1.10 INR ISI
Serum iron	27 μg/dL	50–175 μg/dL
GOT	9.00 UI/L	10–37 UI/L
GPT	6.5 UI/L	12–63 UI/L
Creatinine	0.55 mg/dL	0.6–1.30 mg/dL
Alkaline phosphatase	49.00 U/L	50–136 U/L

## Data Availability

The materials described in the manuscript, including all relevant raw data, will be freely available to any scientist wishing to use them for non-commercial purposes, without breaching participant confidentiality.
